# Non-convulsive status epilepticus as a cause of delayed emergence after a thoracic surgery: a case report

**DOI:** 10.1186/s40981-025-00790-z

**Published:** 2025-05-29

**Authors:** Yusuke Iritani, Makiko Tani, Shinji Iga, Hiroshi Morimatsu

**Affiliations:** 1 Department of Anesthesiology, Okayama Red Cross Hospital, 2-1-1, Aoe, Kita-Ku, Okayama, 700-8607 Japan; 2 Department of Anesthesiology and Resuscitology, Graduate School of Medicine Dentistry and Pharmaceutical Sciences, Okayama University, 2-5-1, Shikata-Cho, Kita-Ku, Okayama, 700-8558 Japan; 3 Department of Anesthesiology and Resuscitology, Okayama University Hospital, 2-5-1, Shikata-Cho, Kita-Ku, Okayama, 700-8558 Japan

**Keywords:** Non-convulsive status epilepticus, Delayed emergence, Anesthesia, Electroencephalography, Postoperative complication

## Abstract

Non-convulsive status epilepticus (NCSE) is an electrical discharge which occurs without prominent motor symptoms. NCSE is one of the causes of delayed emergence from anesthesia; however, as far as we know, previous reports of postoperative NCSE were related to patients after neurological surgery. Herein, we report a case of an elderly male who developed initial NCSE after thoracic surgery. The patient remained unresponsive and developed hemiplegia after lung resection, and then the symptoms fluctuated between better and worse. Metabolic disorders and stroke were ruled out, and NCSE was diagnosed by magnetic resonance imaging (MRI) and electroencephalography (EEG). NCSE occurred in a patient who had no predisposing factors or underwent non-neurological surgery. When anesthesiologists encounter delayed emergence, NCSE should be listed as a differential diagnosis and examined by MRI and EEG.

## Introduction

Non-convulsive status epilepticus (NCSE) is defined as a status epilepticus with cognitive or behavioral changes but without prominent motor symptoms associated with continuous paroxysmal electrographic discharges on the electroencephalography (EEG) [1].

Common symptoms of NCSE are altered consciousness status, speech impairment, myoclonia, and disturbed behavior [2]. Various factors have been reported to be associated with NCSE, including hypoxic-ischemic enchephalopathy, metabolic disorders, stroke, malignancy, infections, drugs, toxins, carbon monoxide, and head trauma [3]. Incidence of NCSE was reported to be 8% in adult impaired conscious patients [4]. Particularly in the emergency departments and the intensive care units (ICU)s, the incidence of NCSE in unconsciousness patients has been reported higher, from 8 to 37% [5–7].

NCSE is known as a common cause of consciousness disorder in the critical care field [5–7]. On the other hand, there have been few reports which reported NCSE as a cause of delayed emergence after general anesthesia in the operating theater [8, 9].

In addition, reports were all regarding patients undergoing intracranial or nerve manipulation which could affect the nervous system. As far as we investigated, there were no previous reports of NCSE which occurred after surgeries other than intracranial or nerve manipulation.

Here, we report a case of delayed emergence due to new-onset NCSE after thoracic surgery in a patient with no particular risk factors.

## Case presentation

We obtained a written consent from the patient for the publication of this case report.

The patient was a 67-year-old male with well-controlled hypertension treated with nifedipine, olmesartan, and azelnidipine. He had undergone partial mandibular resection for adenocarcinoma under general anesthesia. He had no history of seizure or psychiatric illness. He was scheduled to have a video-assisted right lung wedge resection for multiple metastatic lung tumors from submandibular gland tumor. His preoperative lab data were unremarkable.

On the day of the surgery, an epidural catheter was inserted in the T7-8 with no problems noted. After a test dose of 30 mg lidocaine with 30 mcg of epinephrine, the patient had no sign of motor blockade or altered mental status. General anesthesia was induced with propofol, remifentanil, and rocuronium. His trachea was intubated with a double lumen tube. We administered cefazoline for surgical site infection prophylaxis. Anesthesia was maintained with target-controlled infusion of propofol at 2–3 mcg/ml and continuous infusion of remifentanil at 0.05 to 0.3 mcg/kg/min and rocuronium. Total dose of fentanyl and 0.2% ropivacaine during anesthesia was 200 mcg and 44 mg, respectively. His mean arterial pressure (MAP) was maintained around 75 mmHg by an intermittent bolus injection of phenirephrine and ephedrine. There was no episode of hypoxia or hypothermia throughout the surgery. The patient state index (PSI) was displayed from 25 to 50 under SedLine®^□^ (Masimo Corporation, Irvine, CA, USA) monitoring without any specific findings of processed electroencephalography (pEEG).

The surgery was completed in 3 h and 11 min, and we discontinued intravenous anesthetics and administered 140 mg of sugammadex for reversal of neuromuscular block. Train-of-four ratio of 1.0 was confirmed. However, the patient did not respond to verbal commands for 30 min. Gradually, the patient breathed spontaneously with adequate tidal volume, but he was still unresponsive to verbal commands. There were no results of hyponatremia, hypercalcemia, hypoglycemia by blood tests. Subsequently, the patient’s left limbs did not move in response to painful stimulus. Suspecting an acute cerebrovascular event, we performed a computed tomography (CT) scan. There were no particular findings, including intracranial hemorrhage in the CT scan. One hour and 45 min later, we diagnosed hemiplegia, and brain magnetic resonance imaging (MRI) was performed to determine the cause of the delayed emergence after anesthesia. However, no findings, such as acute stroke, intracranial bleeding or brain tumor, were indicated in the MRI. The patient was transferred to the ICU for treatment to delayed emergence from anesthesia. Afterward, his conscious level and left hemiparesis gradually improved, and his trachea was extubated. However, his consciousness level and hemiparesis fluctuated between better and worse repeatedly under no anesthetic agents.

**Fig. 1:**
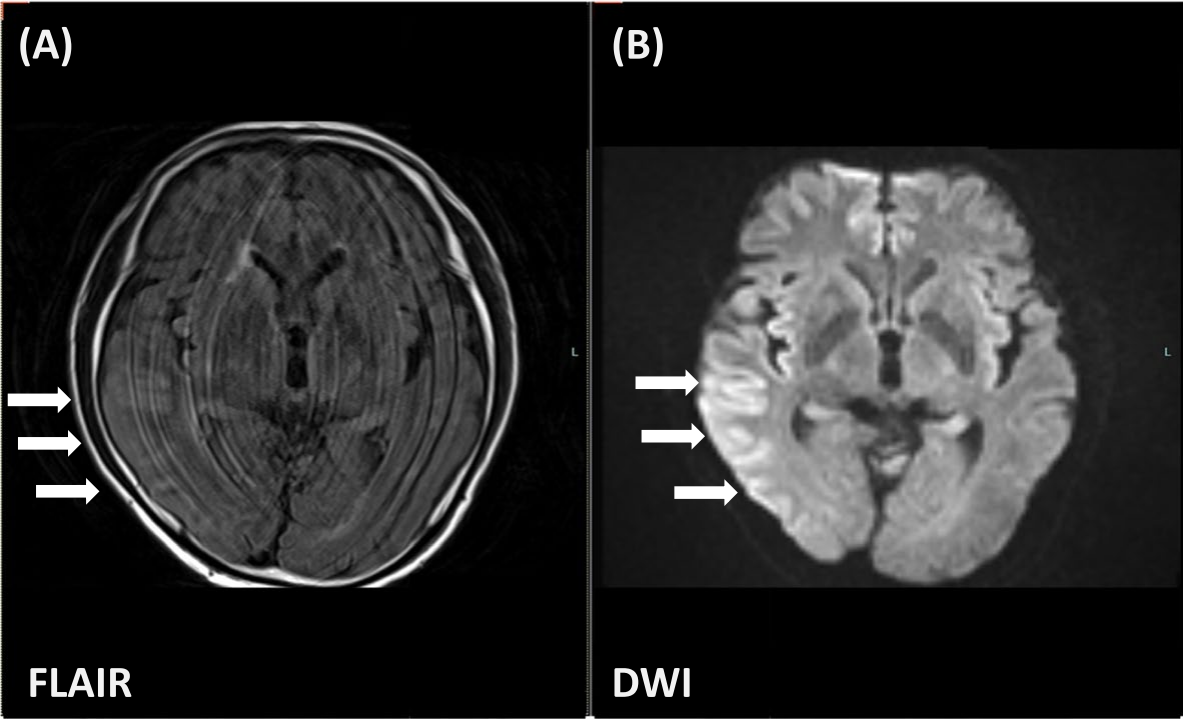
Magnetic resonance imaging of the head on postoperative day 1. Gray matter predominant high signal range over the right cerebral hemisphere. **A** Diffusion weighted image (DWI). **B** Fluid-attnuated inversion recovery (FLAIR)

**Fig. 2:**
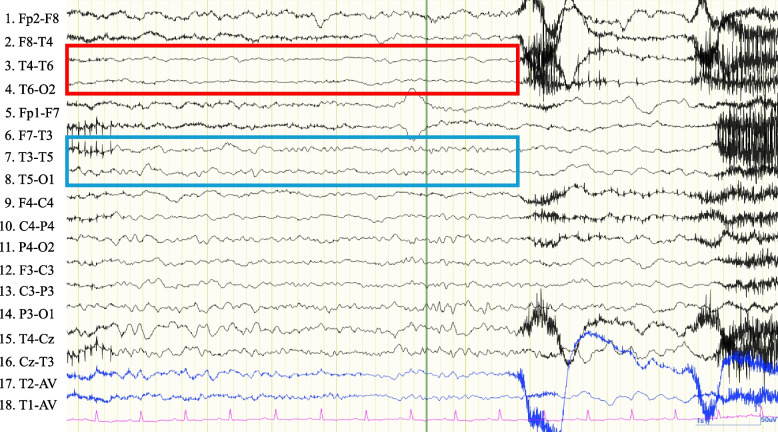
Electroencephalograph on postoperative day 1. Amplitude attenuation of electroencephalography activity with right occipital predominance (waveform surrounded by red square compared to that surrounded by blue square), which is consistent with the high-signal area on the head magnetic resonance imaging

At post operative day 1 (POD 1), an MRI was performed again and indicated gray matter predominant diffuse high signal range over the right cerebral hemisphere in diffusion weighted imaging and fluid-attnuated inversion recovery (FLAIR) images (Fig. 1). Neither organic abnormalities on MRI nor abnormal findings in motor cortex were revealed. In addition, EEG was performed on the same day as scheduled, which showed attenuation of EEG activity with right occipital predominance, demonstrating interhemispheric asymmetry (Fig. 2). These findings of MRI and EEG were thought to be consistent with post-epileptic change mainly in right occipital lobe.

Based on his fluctuating neurological symptom of absence of seizures and left-sided hemiparalysis, along with the results of the MRI and EEG, he was diagnosed with NCSE and Todd’s paralysis, triggered by NCSE. Despite having started the treatment with levetiracetam, a generalized tonic–clonic convulsion occurred, and the patient was reintubated. On POD 2, we added 100 mg of lacosamide to the levetiracetam as a treatment of status epilepticus. His consciousness stabilized, and he showed no apparent convulsion or paralysis. He was extubated again on POD 5 and discharged from the intensive care unit on POD 8. Afterward, his postoperative course progressed well without any neurological complications, and his follow-up MRI and EEG results were normal. A retrospective intraoperative pEEG review showed no findings of NCSE.

## Discussion

This is a case of NCSE diagnosed through delayed emergence from general anesthesia for thoracic surgery that did not affect the nervous system, in a patient with no predisposing factors.

Delayed emergence from general anesthesia is a state of inadequate level of consciousness 30–60 min after the end of general anesthesia due to various causes [10]. The common causes of postoperative delayed emergence include pharmacokinetic causes, pharmacodynamic causes, metabolic alterations, neurological disorders, and psychiatric causes [10]. NCSE is one of the neurological causes of delayed emergence; however, its incidence has not been revealed because of its characteristics which are not diagnosed unless suspected.

In this case, we ruled out common causes of the remaining effect of anesthetics, prolonged effect of neuromuscular blocking agents, and metabolic disorders, such as hypoglycemia and hypothermia. There were no episodes of hypoxemia or prolonged hypotension which could lead to brain hypoxia or hypoperfusion. The CT scan showed no brain ischemia or intracranial bleeding. The patient had no history of conversion disorder. As a result, NCSE could be cited as a differential diagnosis of delayed emergence.

NCSE is defined as a Status Epilepticus not accompanied by prominent motor phenomena [11]. It is reported that NCSE should be suspected when unexplained consciousness disorders and their symptoms are transformative. In this case, we ruled out common causes of delayed emergence, and then we determined the diagnosis of NCSE by MRI and EEG. In our case, the patient had no evident pathogenesis, such as central nerve inflammation, stroke, head trauma, brain tumor, or metabolic disorders.

There have been four case reports published regarding postoperative delayed emergence caused by NCSE. In these case reports, all patients had intracranial surgeries [8, 12, 13] with the exception of one patient who had spine surgery^9)^. In these previous reports, the causes of NCSE were thought to be enigmatic and multifocal with pharmacological roles associated with anesthetic agents or medication during a craniotomy that could alter the blood–brain-barrier [13]. Furthermore, the reports mentioned the importance of EEG measurements to diagnose NCSE.

An EEG is indispensable to diagnose NCSE; however, an intraoperative pEEG monitor (SedLine®) was not effective to detect epileptiform discharges. As far as we had investigated, there was no previous report which described the effectiveness of intraoperative pEEG monitoring. Currently, pEEG is thought to be insufficient to diagnose intraoperative epilepsy. If a consciousness disorder persists regardless of a general workup of delayed emergence, such as electrolyte disorders, stroke, and excessive opioids, a raw EEG should be examined to diagnose or to exclude NCSE. An intraoperative sedation monitor such as Sedline® was not able to detect any EEG abnormality.

MRI could be a useful option for diagnosing NCSE, because MRI has superiority than EEG regadring on less subjective interpretation, 24-h availability, special resolution, and so on [14]. However, we insist that MRI and EEG should be used complementary to the diagnosis of NSCE. First, depending on the timing of MRI imaging, NSCE could not be diagnosed. It is reported that abnormalities on MRI appear during the ictus and up to 11 days post-ictal (median time is 24 h) [15]. In this case, we took MRI right after detecting delayed emergence; however, we could not find any laterality even in the findings of the diffusion weighted image, and we could not specifically identify any left–right differences even in the findings of the diffusion weighted image, and we could not reach the diagnosis of NCSE. Second, there have been some MRI abnormalities in NCSE reported (such as restricted diffusion) [15]; however, current criteria for diagnosing NCSE does not include MRI findings. EEG is indispensable for NCSE diagnosis. Third, MRI imaging requires the removal of substances that produce magnetism and is not easily performed on post-operative or intensive care patients who have many medical devices attached. In addition, such patients require hypnotics to stay calm for taking good-quality MRI. Hypnotics does not matter to detect post-ictal MRI change of NCSE; however, limited to our case, it was necessary to avoid using hypnotics because the primary problem of this patient was delayed emergence.

Regarding on the cause of NCSE in this case, we could not make a specific diagnosis. It cannot be ruled out that the patient had an intraoperative transient ischemic attack and subsequent seizure due to relative hypotension, but was unaware of it due to the effects of anesthetics and muscle relaxants. In addition, there have been some reports of propofol causing epilepsy in the past [16], and there is a possibility that propofol caused NSCE in this case.

In conclusion, we experienced a case of delayed emergence due to NCSE which occurred in a patient who had thoracic surgery. NCSE could be a cause of delayed emergence even after neurosurgeries. It might be impossible to diagnose NCSE using an intraoperative pEEG monitor. Anesthesiologists should consider NCSE as a potential cause of delayed emergence even if there are no abnormal findings on the intraoperative processed EEG, and must investigate raw EEG as soon as possible.

## Abbreviations

NCSE: Non-convulsive status epilepticus
EEG: Electroencephalography
ICU: Intensive care unit
MAP: Mean arterial pressure
PSI: Patient State Index
pEEG: Processed electroencephalography
CT: Computed tomography
MRI: Magnetic resonance imaging
POD: Postoperative day
FLAIR: Fluid-attenuated inversion recovery
